# An Analysis of Semicircular Channel Backscattering Interferometry through Ray Tracing Simulations

**DOI:** 10.3390/s22114301

**Published:** 2022-06-06

**Authors:** Niall M. C. Mulkerns, William H. Hoffmann, Ian D. Lindsay, Henkjan Gersen

**Affiliations:** 1 H. H. Wills Physics Laboratory, University of Bristol, Bristol BS8 1TL, UK; niall.mulkerns@bristol.ac.uk (N.M.C.M.); w.hoffmann@bristol.ac.uk (W.H.H.); i.d.lindsay@bristol.ac.uk (I.D.L.); 2 Bristol Centre for Functional Nanomaterials, University of Bristol, Bristol BS8 1TL, UK; 3 School of Chemistry, University of Bristol, Bristol BS8 1TS, UK

**Keywords:** backscattering interferometry, refractive index, semicircular channel, ray tracing, microfluidics

## Abstract

Recent backscattering interferometry studies utilise a single channel microfluidic system, typically approximately semicircular in cross-section. Here, we present a complete ray tracing model for on-chip backscattering interferometry with a semicircular cross-section, including the dependence upon polarisation and angle of incidence. The full model is validated and utilised to calculate the expected fringe patterns and sensitivities observed under both normal and oblique angles of incidence. Comparison with experimental data from approximately semicircular channels using the parameters stated shows that they cannot be explained using a semicircular geometry. The disagreement does not impact on the validity of the experimental data, but highlights that the optical mechanisms behind the various modalities of backscattering interferometry would benefit from clarification. From the analysis presented here, we conclude that for reasons of ease of analysis, data quality, and sensitivity for a given radius, capillary-based backscattering interferometry affords numerous benefits over on-chip backscattering interferometry.

## 1. Introduction

Backscattering interferometry (BSI) is an optical technique that has found extensive use in the field of biochemical analysis as a simple, robust instrument to measure protein binding and enzyme kinetics in solution [1,2,3]. It has also been used as a sensor for high-performance liquid chromatography [4], electrophoresis [5,6], polarimetry [7], and nanoparticle characterisation [8]. Since its inception, the optical configuration of BSI has changed dramatically. Initially, BSI was performed using a standard glass capillary [9]; however, more contemporary BSI experiments have been performed using an etched channel in a microfluidic chip [10]. Furthermore, the type of channel cross-section has varied from rectangular [11] to approximately semicircular (with light incident from both above [10] and below [12]), with the most recent literature suggesting a return to the capillary-based system [13]. For the capillary-based BSI system, multiple papers exist that generate simulated data utilising both commercial and analytical ray- and wave-based models [7,14,15,16,17]. For the microfluidic chip-based system however, to the best of the authors’ knowledge, only one (wave-based) analytical model exists [17]; others have been developed using commercial software [11,18], leading to difficulty when seeking comparison. However, in the wave-based model, the propagation direction of the incident light is at odds with that used in many subsequent BSI experiments. In addition, attempts at creating a model for a similar BSI geometry (i.e., rectangular channels) have been unsuccessful, with experimental results that seem to defy simulated data [11]. Therefore, an in depth model of on-chip BSI with a semicircular cross-section would be beneficial to understanding recent results obtained using this technique.

In this paper, an analytical model based on ray tracing [19] is outlined that fully describes the expected interference pattern created for BSI performed on a microfluidic chip with a semicircular cross-section. This geometry is relevant to the community as recent literature presents the on-chip channels as having approximately semicircular cross-sections, with a slightly elongated central section due to etching effects [18]. The model presented fully describes the potentially infinite ray types that contribute to the final interference pattern, including polarisation dependence. The model is then verified based on specific cases, and compared and contrasted to previously published experimental results, ultimately finding that experimental results do not correspond to those expected for a semicircular geometry. A number of suggestions of the origin of the discrepancy are presented; the most likely reason being discrepancies in geometries. An overall suggestion on the optimal modality of BSI to use is given, from a theoretical standpoint.

## 2. Ray Tracing Simulations

**Figure 1:**
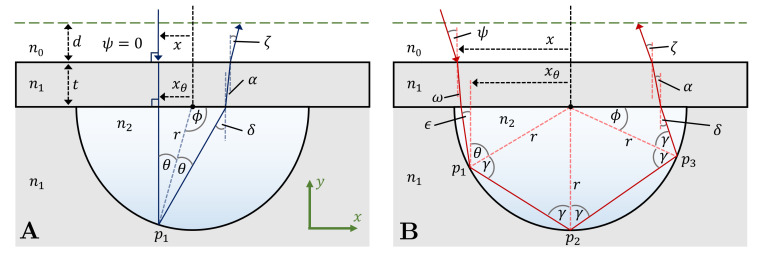
(**A**): A diagram showing the ray path taken when light is incident perpendicular to a chip with a semicircular channel with intersection number i=1. (**B**): The path of a ray when the incident light is oblique to the chip surface with intersection number i=3. Positive *x* is defined to be to the right.

The profile of the microfluidic chip in a typical on-chip BSI experiment is shown in Figure 1. For a schematic showing the full experimental implementation of BSI, the reader is directed to the work of Kammer et al. [10] and Mulkerns et al. [19]. In cross-section, the chip consists of three distinct pieces: a base, a cap, and the enclosed fluid. The base, consisting of an infinitely thick flat plane with a semicircular groove of radius *r*, has a refractive index of $n_{1}$, and is typically fashioned from glass or polydimethylsiloxane. The channel is formed by sealing the base with a rectangular cap of some thickness *t* and will contain the fluid of interest with refractive index $n_{2}$. In the majority of the literature, the same material of refractive index $n_{1}$ is used for the cap as for the base; this convention will be echoed here. The chip itself is surrounded by a medium of refractive index $n_{0}$, typically assumed to be air. The light incident on the channel is considered to be a plane wave of wavelength λ at some angle ψ to the surface normal, causing the irradiance at any point on the surface to be constant. As the channel is symmetric about the origin (denoted by a black dot in Figure 1), the problem can be simplified by considering one side of the channel in the case of normally incident light, but this symmetry is broken under oblique illumination. The general case of non-perpendicular incident light is considered here; however, special attention is paid to the special case of ψ=0 which is typically used in BSI.

As ray tracing is a deterministic technique, the propagation of a light ray through the system can be fully calculated by geometrical arguments. It should be noted that effects such as diffraction can not be simulated using ray tracing; however, in the case analysed here, the length scales probed are much larger than the wavelength of light used, and as such, geometrical optics is appropriate. First, the angle of each ray at a given interface is elucidated, which can be used to determine the path lengths as well as the relative amplitude and phase of each ray. In addition, the bounds for which a given ray is actually defined can be determined by considering these angles. From this, the final fringe pattern at a given position is calculated by interfering the appropriate rays.

### 2.1. Angular Bounds

Consider a ray that is incident on the surface at some angle ψ that intersects the cap at some point *x* away from the origin. In this manuscript, left is defined to be negative *x* and right positive, with x=0 at the origin of the radius of the channel, as shown in Figure 1. The incident angle of light is confined to be between 0≤ψ≤π/2. In the special case that x=0 and ψ=0, the reflected ray will travel back along the same path it entered through. However, if x≠0 and ψ=0 (for example, see Figure 1A), then the ray will intercept the channel circumference at $x=x_{\theta }$ and some angle θ which is described by

(1) $$\theta =\arcsin(-x_{\theta }/r).$$

However, in general (i.e., when ψ≠0), *x* and $x_{\theta }$ are not the same but are linked by

(2) $$x_{\theta }=x+t\tan\left(\omega \right)+r\cos\left(\theta \right)\tan\left(\epsilon \right),$$

where $\omega =\arcsin[n_{0}/n_{1}\sin\left(\psi \right)]$, *t* is the thickness of the cap, and the other parameters as defined in Figure 1. As θ is dependent on $x_{\theta }$, Equation (2) can be fully solved in terms of $x_{\theta }$. This form, though less intuitively understandable, is given in the Supporting Information.

Depending upon the magnitude of θ and the incident angle of the light ψ, the ray will go on to intersect the radius of the channel *i* times, with *i* henceforth referred to as the intersection number of a given ray. In this case, at very small *x* values, the intersection number will be 1, but as x→r, the number of intersections will approach *∞*. In the case of ψ=0, the ray will be reflected from the channel surface at an angle θ, however in the case that ψ≠0, the ray will be reflected at an angle

(3) γ=θ+ϵ,

as shown in Figure 1B. Here ϵ can be found by repeated usage of Snell’s law.

The relationship between the intersection number and the incident position of the light ray can be elucidated by considering the value of ϕ as shown in Figure 1. Taking the example of the ray in Figure 1B, as the incident position of the ray moves towards the origin, the intersection point $p_{3}$ will move closer to the cap until ϕ=0 and $p_{3}$ ceases to exist. For some intersection number *i*, ϕ can be determined by summing the angles around the origin:

(4) $$\phi =\frac{\pi }{2}\left(3-2i\right)+2\gamma \left(i-1\right)+\theta .$$

As ϕ>0 for a given *i*, rearranging leads to

(5) $$i>\frac{3/2\pi -2\gamma +\theta }{\pi -2\gamma },$$

which sets the bounds on the angles (and therefore the *x* values given a known ψ value) that cause a ray with *i* intersections. Equation (5) defines a continuous function, but the number of intersections of the ray with the channel can only be a natural number. Therefore, it should be amended to

(6) $$i=\left\lfloor \frac{3/2\pi -2\gamma +\theta }{\pi -2\gamma }\right\rfloor ,$$

where ⌊⌋ denotes the floor function. Equation (6) can be seen to give the correct values of i=1 at θ=0 (i.e., x=0) and i→∞ as θ≈γ→π/2 (i.e., x→±1) at normal incidence. The bounds for the first 6 intersection numbers for the case of normal incidence (ψ=0) are given in Table 1.

By considering the right-angled triangle formed by the final intersection point and the origin, the angle of any ray that strikes the underside of the cap will always do so at an angle of δ, where

(7) $$\delta =\frac{\pi }{2}-\phi -\gamma .$$

By application of Snell’s law, the angle of the ray exiting the cap of the channel will be

(8) $$\alpha =\arcsin\left(\frac{n_{2}}{n_{1}}\sin\left(\delta \right)\right).$$

As $n_{1}$ is typically higher than $n_{0}$, some of these rays will strike the cap-air boundary at angles greater than the critical angle $\alpha_{crit}=\arcsin\left(n_{0}/n_{1}\right)\approx 43.2^{\circ }$ (for $n_{1}=1.46$, $n_{0}=1$). By combining Equations (7) and (8), for a given intersection number *i* the minimum and maximum allowed x/r values are given implicitly by

(9) $$\phi +\gamma =\arccos\left(\frac{n_{1}}{n_{2}}\sin\left(\pm \alpha_{crit}\right)\right).$$

By substituting Equation (4) into Equation (9) and rearranging, in the special case that ψ=0, the range of x/r values that lead to totally internally reflected rays are given by

(10) $${\left(\frac{x}{r}\right)}_{TIR}=\sin\left(\frac{1}{2i}\left[\arccos\left(\frac{n_{1}}{n_{2}}\sin\left(\pm \alpha_{crit}\right)\right)+\left(2i-3\right)\frac{\pi }{2}\right]\right).$$

Thus, for ψ=0, x/r values between 0.431 and 0.542 (correct to 3 decimal places) will not contribute to the final fringe pattern seen.

### 2.2. Path Lengths

As the angles and intersection number of a given ray are now defined, the optical path length of the ray between each interface can be evaluated [20]. By calculating the optical path length of a given ray, the phase difference between the rays can be determined when they are interfered.

**Figure 2:**
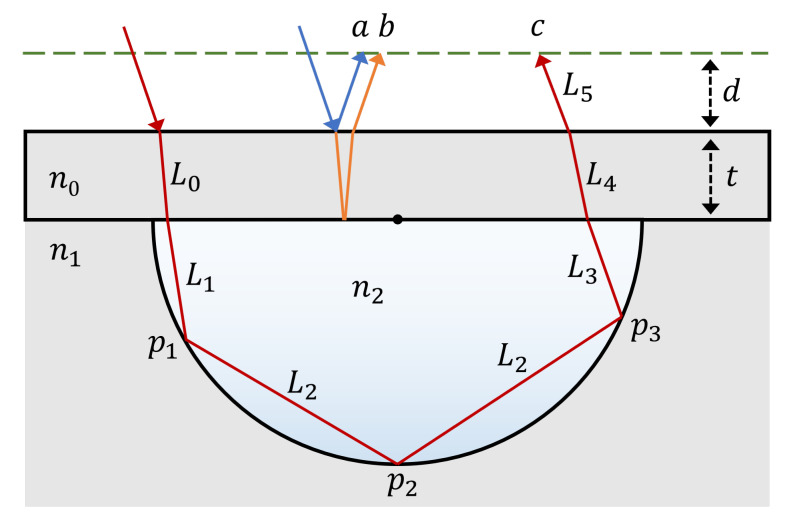
A diagram showing examples of a type *a*, *b*, and *c* ray, highlighting the segments of the *c* ray that correspond to the path lengths sections $L_{0-5}$ as described in the text.

For clarity, each incident ray can be split into three distinct sub-rays labelled *a*, *b*, and *c*, as shown in Figure 2. Rays *a* and *b* are the rays that reflect from the top and the bottom of the cap respectively. Ray *c* is defined to be any ray that intersects with the channel radius, no matter how many times.

Taking first the general case of a *c*-type ray with intersection number *i*, the optical path length can be split into 6 separate parts, as shown in Figure 2. The path length of the light from the top surface to the first intersection with the channel radius is given by

(11) $$L_{0}=n_{1}\left(\frac{t}{\cos(\omega )}\right)$$

and

(12) $$L_{1}=n_{2}\left(\frac{r\cos(\theta )}{\cos(\epsilon )}\right),$$

where all the symbols have the same definitions as previously described. From the first intersection point $p_{1}$ to the final intersection point $p_{i}$, the path length is simply the sum of the base lengths of the isosceles triangles between consecutive points. Therefore, the subsequent path length contribution can be written as

(13) $$L_{2}=n_{2}\left[2r(i-1)\cos(\gamma )\right].$$

In the case that i=1, $L_{2}=0$ as no paths between intersection points are created. The next part of the path length from the final intersection of the ray with the channel to the cap is given by $L_{3}$

(14) $$L_{3}=n_{2}\left(\frac{r\sin(\phi )}{\sin(\phi +\theta )}\right).$$

In the special case that i=1, ϕ=π/2+θ and so $L_{3}$ describes the optical path length of the ray between the only intersection point $p_{1}$ and the cap. Lastly, the path inside the cap and from the cap to the imaging plane can be written as

(15) $$L_{4}=n_{1}\left(\frac{t}{\cos(\alpha )}\right)$$

and

(16) $$L_{5}=n_{0}\left(\frac{d}{\cos(\zeta )}\right),$$

respectively, where *d* is the distance from the cap-air interface to the imaging plane and ζ is the final exit angle of the ray from the chip given by

(17) $$\zeta =\arcsin\left(\frac{n_{1}}{n_{2}}\sin\left(\alpha \right)\right).$$

The final path length of ray *c* is given by

(18) $$L_{c}=\sum_{i=0}^{5}L_{i}.$$

For rays *a* and *b*, the formulae are simpler. The optical path lengths for these rays are given by

(19) $$\begin{array}{cc} L_{a} & =n_{0}\left(\frac{d}{\cos(\psi )}\right) \end{array}$$

(20) $$\begin{array}{cc} L_{b} & =n_{0}\left(\frac{d}{\cos(\psi )}\right)+n_{1}\left(\frac{2t}{\cos(\omega )}\right), \end{array}$$

which for a fixed input angle ψ are independent of *x*. No path lengths between the source and the cap are considered for any ray types as this is the same for all rays due to only rays of a single angle being considered. However, this constraint could be relaxed if needed, such as to facilitate simulation of a focused beam.

### 2.3. Intensity Calculations

To calculate the fringe pattern seen, all that is left to calculate is the relative amplitude of each ray that arises for a given incident angle and position of the illumination source.

A ray of fully *s*- or *p*-polarised light incident on a surface at some angle *A* and reflected/transmitted at some angle *B* will have amplitude reflection and transmission coefficients defined by the Fresnel equations. In terms of *A* and *B* these are

(21) $$\begin{array}{cc} R_{s} & =\frac{\sin(A-B)}{\sin(A+B)}, \end{array}$$

(22) $$\begin{array}{cc} T_{s} & =\frac{2\cos(A)\sin(B)}{\sin(A+B)}, \end{array}$$

for *s*-polarised light, whereas for *p*-polarised light they are

(23) $$\begin{array}{cc} R_{p} & =\frac{\tan(A-B)}{\tan(A+B}), \end{array}$$

(24) $$\begin{array}{cc} T_{p} & =\frac{2\cos(A)\sin(B)}{\sin(A+B)\cos(A-B)}. \end{array}$$

where the polarisation is not specifically stated, *R* and *T* will be used to denote the polarisation dependent versions $R_{s/p}$ and $T_{s/p}$. In general, *B* can be determined using Snell’s law. It is pertinent to note that *R* and *T* are here defined in terms of amplitude, not power, to allow inclusion of the phase changes of the ray upon reflection/transmission. In addition, the shorthand of, for example, $R(A,n_{A},n_{B})$ is used to denote the reflection coefficient of a ray of angle A travelling in a medium with refractive index $n_{i}$ incident on a medium of refractive index $n_{B}$.

It is possible to determine the relative amplitude of the rays from every x/r value by following the path taken through the system and applying the correct reflection or transmission coefficient. In this paper, the incident intensity of the light is always $I_{0}=1$, though it would be possible to substitute it for any other factor or indeed one that is a function of x/r (e.g., a Gaussian beam). For any ray, the first two interfaces will give rise to two reflected components from the interfaces encountered (*a* and *b*), as well as a transmitted ray (*c*) that will continue on, as seen in Figure 2. In general, a *b*-type ray will be approximately 10× lower in intensity than an *a* ray for the materials typically used in on-chip BSI. The rays that continue on will strike the circumference of the channel at an angle γ a number of times equal to *i*. This leads to an extra factor of $R{\left(\gamma ,n_{2},n_{1}\right)}^{i}$ if it is assumed that the material of the cap is the same as the channel material. Lastly, the ray must transmit back through the cap to be detected, leading to the final factors of $T\left(\delta ,n_{2},n_{1}\right)T\left(\alpha ,n_{1},n_{0}\right)$. Therefore, for a single x/r value with a corresponding intersection number *i*, a maximum of three different rays will be created with relative amplitudes of

(25) $$\begin{array}{cc} s_{a} & =R(\psi ,n_{0},n_{1}) \end{array}\;\;\;\;\;\;\;\;\;\;\;\;\;\;\;\;\;\;\;\;\;$$

(26) $$\begin{array}{cc} s_{b} & =T\left(\psi ,n_{0},n_{1}\right)\;\cdot \;R\left(\omega ,n_{1},n_{2}\right)\;\cdot \;T\left(\omega ,n_{0},n_{1}\right) \end{array}\;\;\;\;\;\;\;\;\;\;\;$$

(27) $$\begin{array}{cc} s_{c} & =T\left(\psi ,n_{0},n_{1}\right)\;\cdot \;T\left(\omega ,n_{1},n_{2}\right)\;\cdot \;R{\left(\gamma ,n_{2},n_{1}\right)}^{i}\;\cdot \;T\left(\delta ,n_{2},n_{1}\right)\;\cdot \;T\left(\alpha ,n_{1},n_{0}\right), \end{array}$$

where $I_{a/b/c}=I_{0}s_{a/b/c}^{2}$. It can immediately be seen that for a given ψ value, $s_{a}$ and $s_{b}$ are constant in magnitude, but $s_{c}$ varies with *x*.

The density of incident rays is equal on the surface of the channel; however, the density of rays reaching a certain location on the viewing plane is not constant. Therefore, equally spaced rays in the imaging plane would not lead to evenly spaced rays in the incident plane, potentially under- or over-sampling rays of certain intersection numbers. To correct for this, a multiplicative factor of *f* must be introduced to each $s_{c}$ value [15,21]. Consider a piece of cap extending from *x* to x+dx in width that has incident upon it *N* rays of intensity $I_{in}$ each, which may be a function of position (here only light that eventually makes it to the detector is considered). As these rays are transmitted and reflected, the extremal rays will be detected at $x^{\prime }=g\left(x\right)$ and $x^{\prime \prime }=g\left(x+dx\right)$, where *g* is some function defining the relation between the input and output *x*-coordinates. It is assumed here that the dx segment is small enough to not contain any discontinuities and that g is not multivalued within the range $x^{\prime }$ to $x^{\prime \prime }$ (i.e., the intersection number is constant). In that case, the number of rays between $x^{\prime }$ and $x^{\prime \prime }$ must also be *N*. If dx is sufficiently small, $x^{\prime \prime }$ can be written as $x^{\prime \prime }=x^{\prime }+\left(dx^{\prime }/dx\right)\;\cdot \;dx$ and so assuming conservation of energy density holds

(28) $$\begin{array}{cc} \frac{\sum^{N}I_{in}}{(x+dx)-x} & =\frac{\sum^{N}I_{in}}{\left(x^{\prime }+\frac{dx^{\prime }}{dx}dx\right)-x^{\prime }}\\ \frac{\sum^{N}I_{in}}{dx} & =\left(\frac{\sum^{N}I_{in}}{dx}\right){\left(\frac{dx^{\prime }}{dx}\right)}^{-1}, \end{array}$$

which is clearly incorrect. This shows that a factor of ${\left(\frac{dx^{\prime }}{dx}\right)}^{-1}$ is required to balance the equation. As the number of rays and area cannot be changed, as a reduction of one would reduce the other, the amplitude of a given ray therefore must be multiplied by a factor

(29) $$f={\left(\frac{1}{\left|\frac{dx^{\prime }}{dx}\right|}\right)}^{1/2}$$

to conserve energy density, where the absolute value is used to ensure intensities cannot be negative.

By considering only the change in the *x* position of the light as it propagates through the chip, the full analytical equation for the output position on the detector plane $x^{\prime }$ in terms of the input position *x* for a type *c* ray is found to be

(30) $$x^{\prime }=x+\tan\left(\omega \right)+r\cos\left(\theta \right)\tan\left(\epsilon \right)+\chi -r\sin\left(\phi \right)\tan\left(\phi +\gamma \right)-t\tan\left(\alpha \right)-d\tan\left(\zeta \right),$$

where χ is defined to be the *x* position change from first to final intersection point within the channel, which is dependent upon the intersection number of the ray. This term is given by

(31) $$\chi =2r\cos\left(\gamma \right)\left[\sum_{k=2}^{i}\sin\left(\theta +\left(2k-3\right)\gamma -\left(k-2\right)\pi \right)\right].$$

To determine the final intensity $I(x^{\prime },d)$ of the fringe pattern at a particular horizontal position $x^{\prime }$ on the image plane at some distance *d*, the rays must be interfered pairwise [14,16]. The total intensity at some position $x^{\prime }$ is given by [17]

(32) $$I\left(x^{\prime },d\right)=\sum_{j=0}^{l}\sum_{k=j+1}^{l}s_{j}\left(x_{j}\right)s_{k}\left(x_{k}\right)\cos\left(\frac{2\pi }{\lambda }\left[L_{j}\left(x_{j}\right)-L_{k}\left(x_{k}\right)\right]\right),$$

where λ is the wavelength of the light and *l* is the total number of rays that can intersect the detector plane at $x^{\prime }$. In Equation (32), $x_{j}$ and $x_{k}$ are defined to be the *x* positions of the incident light that give rise to an output position $x^{\prime }$ for the *j* and *k* rays. The $s_{j}$ and $s_{k}$ terms here should each include the factor of *f* as set out in Equation (29) for their respective incident positions if appropriate.

By considering a detection plane some distance *d* away from the surface of the chip, evenly spaced points at which to evaluate the intensity can be chosen to simulate the pixels of a camera. From this, the incident position of rays that give rise to components that would all intersect the detector plane at some position $x^{\prime }$ can be determined using Equation (30). These rays can then be interfered pairwise using Equation (32) and the interference pattern determined. If not otherwise stated, the values used in the following simulations are $n_{0}=1.0$, $n_{1}=1.46$, $n_{2}=1.33$, r=100 μm, t=1100 μm, λ=632.8mn, d=1 m, and $x_{w}^{\prime }=13.1\mathrm{mn}$, where $x_{w}^{\prime }$ is the width of the detector used here.

## 3. Validation of Model

**Figure 3:**
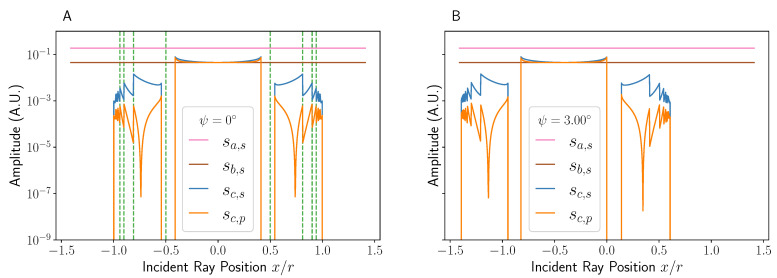
Graphs showing the relative amplitudes (where $I_{0}=1$) of type *c* rays for both *s*- and *p*-polarised incident light at normal incidence ($\psi =0^{\circ }$, (**A**)) and oblique incidence ($\psi =3^{\circ }$, (**B**)). The data here are taken using the standard parameters as defined in the main text. Values of x/r between ±1.5 are simulated to sample the full range of values that give rise to type *c* rays. The dashed lines in A represent the bounds on a given intersection number, with the central section denoting i=1 and increasing by 1 upon crossing a line moving outwards. The sudden reduction in amplitude at the edges is due to the rays no longer entering the channel at this angle. The amplitudes of type *a* and *b* rays for *p*-polarised light are omitted due to their similarity with their *s*-polarised counterparts.

Before using the model developed here for physical predictions, it must be verified to yield sensible data in the case of both normal and oblique illumination. The amplitude of the rays created as a function of incident ray position are shown in Figure 3 for both illumination cases; this is equivalent to solving Equations (25)–() for each x/r value. It can be seen that in the case of normal illumination (Figure 3A), the final amplitude is symmetrical about x/r=0 as would be expected. In addition, the lines in Figure 3A denoting the change of intersection numbers perfectly align with the discontinuities in the amplitude, as one would expect due to the addition of another factor of $R(\gamma ,n_{2},n_{1})$ (see Equation (27)). On the other hand, Figure 3B shows that a small increase in incident angle is equivalent to shifting the graph for normal incidence to the left. This is intuitive as the rays will now have a small positive displacement in *x*, as can be seen in term 2 of Equation (30), meaning that their $x_{\theta }$ will no longer be equal to *x* and so appear to come from a position further to the right. The graphs also show how the *p*-polarised incident light leads to much lower amplitudes across the x/r range, other than at near normal incidence, which is expected from the Fresnel coefficients. The drop off at x/r≈0.75 in Figure 3A for *p*-polarised light is due to $\theta \to \theta_{B}$, where $\theta_{B}=\arctan\left(n_{2}/n_{1}\right)\approx 47.6^{\circ }$ is the Brewster angle.

Of particular note in Figure 3 is the relative average intensities of each intersection number. In amplitude, the lowest and highest intersection number rays differ by a factor of ∼×30 for *s*-polarised light, and by a factor of ∼×300 for *p*-polarised light. As the type *a* and *b* rays have magnitudes of ∼0.2 and ∼0.045, respectively, at all x/r values, this means that the *c* type ray will be the lowest intensity component of any pair of interference terms.

**Figure 4:**
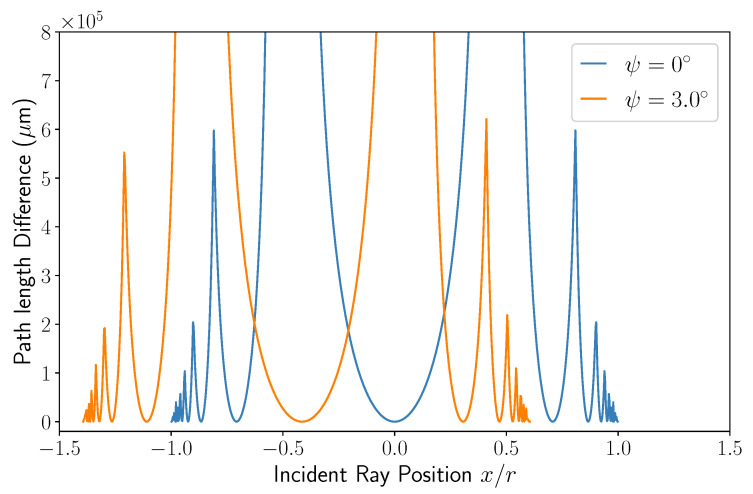
A graph showing the optical path length difference of a type *c* ray for both normal and oblique incidence ($0^{\circ }$ and $3^{\circ }$, respectively). The difference is defined to be with respect to a ray of ψ=0 and x=0 (i.e., solving Equation (18) and subtracting $2n_{2}r+2n_{1}t+n_{0}d$). Data were simulated using the parameters as set out in the main text.

The data for the difference in path length of type *c* rays as a function of incident position x/r, shown in Figure 4, presents similar features as the amplitudes in Figure 3. The difference here is defined to be with respect to the minimum path length (i.e., $L_{c}=2n_{2}r+2n_{1}t+n_{0}d$ when x=ψ=0). There are asymptotes at the points where the light cannot escape the channel due to total internal reflection, as mentioned in the theory section, and sharp local spikes where the intersection number changes. The data for the oblique illumination case (ψ≠0) also show the same translation compared to the normal incidence data in Figure 3, as expected. As both the path lengths and amplitudes of the rays at all input positions have been scrutinised and found to follow expectations, the intensity of the fringe pattern can be determined and analysed with confidence.

## 4. Interference Patterns

### 4.1. Normal Incidence

**Figure 5:**
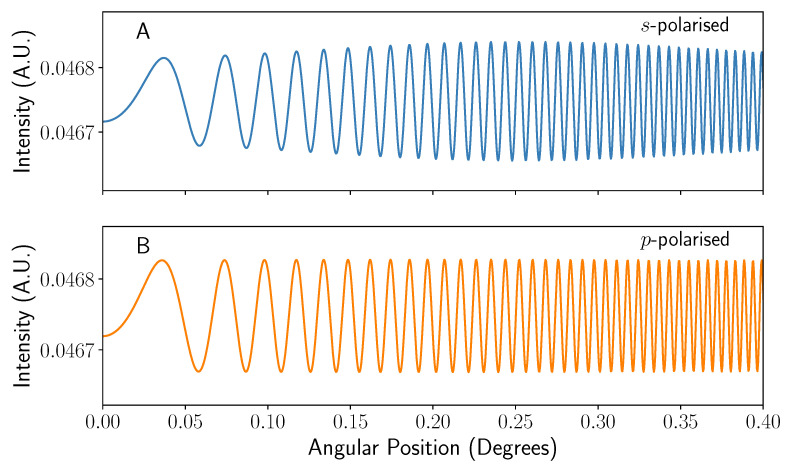
Graphs showing the interference patterns seen on a detector using the parameters as set out in the main body of text at a distance of 1m with the angle given from the line of x=0. (**A**) shows the interference pattern for *s*-polarised incident light, whereas (**B**) shows the pattern imaged for *p*-polarised light.

In the vast majority of literature, the light is incident perpendicular to the capillary or channel [10,11]. Therefore, it would be best to first analyse the simulation created here for a value of ψ=0. The interference patterns as seen by the detector starting at the $0^{\circ }$ backscatter direction at a distance 1 m from the chip and channel radius of r=100 μm can be seen in Figure 5. The data show that in essence both *s*- and *p*-polarised light give rise to a two beam interference pattern, consisting of the interference between *a*-, *b*-, and *c*-type rays where i=1. The data for *s*-polarised light show a slight, low frequency modulation term due to the influence of i=2 rays that are negligible when using *p*-polarised light (see Figure 3). The low modulation depth (i.e., peak to trough intensity difference) of the fringes is expected given the respective amplitude of the i=1*c*-type rays to the *a*- and *b*-type, as shown in Figure 3. The mean value of the fringe pattern pattern intensity (i.e., the baseline) is dictated primarily by the interference between the *a* and *b* rays—specifically by their phase difference. This phase difference is entirely controlled by the path length difference ($L_{b}-L_{a}=2tn_{1}$ for normally incident light), and so by manipulating the thickness of the cap on the nanometre scale, the phase difference and therefore baseline intensity can be tuned, for example, to observe the small variations without detector saturation.

**Figure 6:**
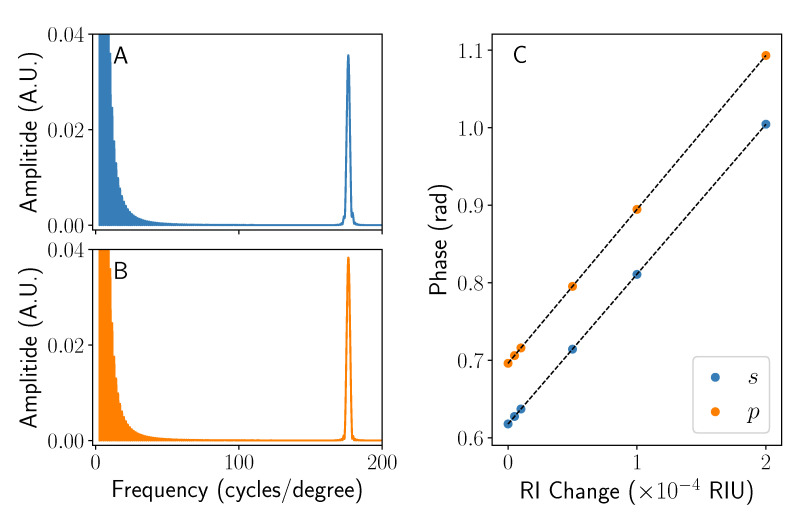
A graph showing the dechirped Fourier transform of the interference pattern seen at normal incidence. A single sharp peak in the Fourier domain is seen here for both *s*- (**A**) and *p*-polarised (**B**) incident light. A graph showing how the phase of each peak in (**A**,**B**) changes as a function of refractive index $n_{2}$ is shown in (**C**). All data were taken using the parameters set out in the main text.

Comparing Figure 5A,B shows that both fringe patterns are fully in phase with each other and show significant increase in spatial frequency as the angular viewing position increases. This spatial frequency change is known as chirping [17,19]. Mathematically, the spatial frequency *f* of the interference increases linearly with position such that

(33) $$f\left(x^{\prime }\right)=f_{0,jk}+\alpha_{jk}x^{\prime },$$

where $\alpha_{jk}$ denotes the “chirp rate”, or spatial frequency increase with position, of the interference between two rays *j* and *k* [17,19]. Equation (33) will still hold when considering angles (ζ) rather than position ($x^{\prime }$), though only in the case of small angles being considered. The chirping causes broadening of the signal when viewed in the Fourier domain, but can be compensated for by determining the correct $\alpha_{jk}$ via spectrographic methods [19] in a process typically referred to as dechirping. By implementing a rolling Fourier transform to create a spectrogram, the rate of instantaneous spatial frequency change with position can be extracted. Using Figure 5, the frequency of both fringe patterns is found to be sharply clustered around f≈170 cycles/degree (see Figure 6A,B). The sharp peaks seen at frequencies close to zero in Figure 6A are an artefact of the windowing and zero padding of the input signal, which is in turn applied to reduce ringing.

In a typical BSI experiment, a series of solutions with varying refractive indices are introduced into the semicircular channel, causing the fringes to be laterally displaced [22]. The position of these fringes can be monitored by tracking the phase Φ of a peak in Fourier space. The sensitivity of the system can be determined by changing the refractive index $n_{2}$ of the fluid in system and noting the phase. This parameter is of great importance as it dictates the limit of detection of the system. For the semicircular channel simulated here, the fringes move laterally by $1.94\times 10^{3}\mathrm{rad}\;{\mathrm{RIU}}^{-1}$ and $1.99\times 10^{3}\mathrm{rad}\;{\mathrm{RIU}}^{-1}$ for *s*- and *p*-polarised light, respectively. This is shown pictorially in Figure 6C. These values (in addition to the phase value at $\Delta n_{2}=0$) can vary a small amount (∼1–2%) both in the simulation and experimentally depending on the choice of Fourier peak position, however the same position was maintained for both polarisation cases here. In addition, large changes in refractive index of the fluid (∼$10^{-4}$) can cause minute shifts in the position of the Fourier peaks, in turn modifying the phase of the chosen position (that is no longer exactly on the peak). Considering this, no errors on the sensitivities are given.

From the sensitivity, it is possible to work out the average optical path length taken by the light in the liquid. The shortest path the light could take through the sample is at x/r=0, being equal to $2rn_{2}$, leading to a phase change per unit refractive index change (i.e., sensitivity) of $d\Phi /dn_{2}=4\pi r/\lambda$. Conversely, the greatest phase change per unit refractive index change is found as x/r→1, where $d\Phi /dn_{2}=2\pi^{2}r/\lambda$. Using the same parameters as used for the simulation in Figure 6, $1.985\times 10^{3}\mathrm{rad}\;{\mathrm{RIU}}^{-1}<d\Phi /dn_{2}<3.119\times 10^{3}\mathrm{rad}\;{\mathrm{RIU}}^{-1}$. Therefore, the sensitivities determined from Figure 6 are dominated by the rays that take the shortest path (i.e., near x/r=0). This is not unexpected, as from Figure 3, the rays where i>1 largely do not contribute to the signal seen, causing the low x/r region to dominate where the average optical path length in the fluid is very close to the smallest possible value.

### 4.2. Oblique Incidence

**Figure 7:**
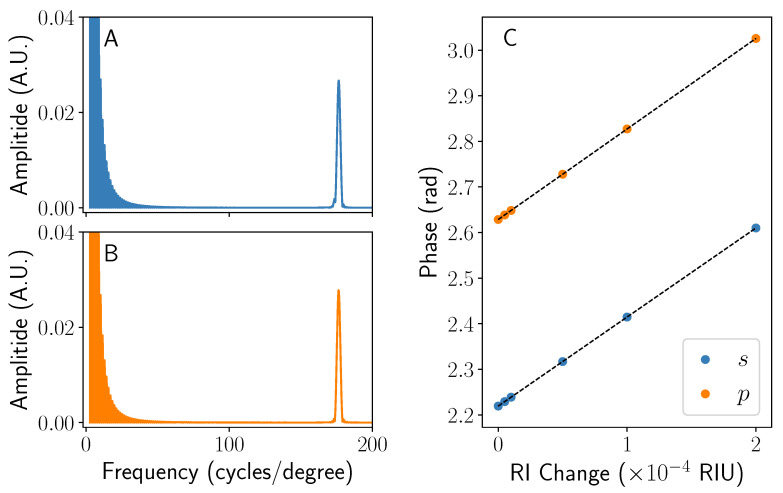
A graph showing the dechirped Fourier transform of the interference pattern seen at an incident angle of $\psi =3{}^{\circ }$. A sharp peak in the Fourier domain as seen in Figure 6 is also seen here for both *s*- (**A**) and *p*-polarised (**B**) incident light. A graph showing how the phase of each peak in (**A**,**B**) changes as a function of refractive index $n_{2}$ is shown in (**C**). All data were taken using the other parameters set out in the main text.

Under illumination at an oblique angle (in this case $\psi =3{}^{\circ }$), the fringe patterns seen are very similar to those in Figure 5 (see Supporting Information). When the same analysis (including dechirping) as described for normal incidence is undertaken for the case where ψ≠0, the Fourier transforms and change in phase with refractive index of $n_{2}$ are shown in Figure 7. The position and clarity of the peaks in Figure 7A,B after dechirping is very similar to their counterparts in Figure 6A,B, which, coupled with the similar fringe patterns, suggests that the interference between each pair of terms varies very little with small values of ψ. Subsequently, the sensitivity of the peaks shown in Figure 7C are found to negligibly different from those in Figure 6C. This is to be expected, as the difference in path length due to the change in ψ will be very small. Physically, these simulations show that the effect of angular misalignment of the chip/laser system has little influence on the final fringe pattern and subsequent phase data.

## 5. Comparison to Literature

After validating and exploring the implications of the model set out in this paper, direct comparison with the results given in the literature will help shed light on chip-based BSI.

It is interesting to note that, for a very similar geometry, Kammer et al. [10] stated a sensitivity of $d\Phi /dT\approx 146\;m\mathrm{rad}\;K^{-1}$, which is equivalent to $d\Phi /dn_{2}=1.38\times 10^{3}\mathrm{rad}\;{\mathrm{RIU}}^{-1}$, if the liquid has the refractive index increment of water, $dn_{2}/dT\approx 1.06E-4\mathrm{RIU}\;K^{-1}$ [10]. Temperature changes may also give rise to refractive index changes in the material the chip is made from, though these are typically lower in magnitude than for water. Temperature-mediated effects on BSI have been discussed in greater detail previously [19]. As the radius of the channel and the wavelength of light used by Kammer et al. is stated to be the same as the parameters employed here, r=100 μm and λ=632.8, the value for $d\Phi /dn_{2}$ found should lie within the bounds stated previously, which is not the case. Assuming the shortest path length dominates as shown by the simulations here ($d\Phi /dn_{2}=4\pi r/\lambda$), the stated sensitivity would instead be commensurate with a channel radius of 69 μm, which differs from the value given by Kammer et al. A similar value is found when analysing the sensitivity given by Kammer et al. using known concentrations of glycerol in phosphate buffer solution (PBS) to change $n_{2}$ [10], suggesting good consistency and reproducibility. We stress that these discrepancies do not in any way negate the results stated by Kammer et al., as their interferometer is fully characterised for phase difference measurements by simply running a calibration with a known compound.

Comparison of the results stated here to literature is difficult due to some of the parameters being undetermined, as well as slight deviations from true semicircularity. When comparing the interference fringes created by the simulation as set out in this paper with those as created by an approximately semicircular channel used in BSI (i.e., that used by Kammer et al. [10]), it is found that the fringe patterns are dissimilar. The fringe frequency is much higher in the simulations examined here (after correcting for the different detector distances), in addition to the modulation depth of the fringes being much greater in the work of Kammer et al. [10]. On the other hand, the single, chirped, frequency of the fringes are consistent between this model and the experimental work. There may be a variety of reasons for this. The most obvious is the discrepancy in geometries; the channel used by Kammer et al. is stated to have a full width of 210 μm and semicircular radius of 100 μm, suggesting that there is a 10 μm approximately flat section in the centre of the channel due to etching effects [18]. As the highest intensity type *c* rays originate from the centre, these rays would likely influence the final fringe pattern dramatically. If one assumes that the chip surface is perfectly flat across the 10 μm segment, any ray that reflects from it will have the same path length, no matter the incident angle of the light. However, if the channel surface were to have some small slope (on the same order of magnitude as the wavelength of light used) in the centre, then that could give rise to a more slowly varying fringe pattern, as seen by Kammer et al. This would cause the system to behave similarly to a Fizeau wedge interferometer [20]. The same effect would also happen if the cap had a small slope, although in this case it is likely that multiple frequency fringes would be seen, with some not being sensitive to changes in $n_{2}$, which cannot be seen in the work of Kammer et al. Additionally, discrepancies may arise from the omission of the ray that passes through the semicircular channel and reflects from the bottom of the chip (i.e., the assumption that the chip is infinitely thick). Comparison would be difficult due to the unknown nature of the substrate on which the microfluidic chip rests, but if suspended in air, this ray may be significant enough in amplitude to warrant inclusion and affect the resultant fringe pattern. Further consideration is needed to evaluate this point, but this is beyond the scope of this paper. Lastly, this potential slope may also explain the inability for models to correctly describe the fringe patterns seen when using rectangular channels [11], which in general should not create interference fringes as there is no varying path length difference. From this, we can conclude that the interpretation of the on-chip BSI system as semicircular does not fully explain the observed fringe patterns.

Compared to BSI performed on a capillary [19,23], the sensitivity of the on-chip variant is approximately halved for a given radius, which is expected when comparing the optical path length through a circle compared to a semicircle. The comparatively high manufacturing tolerances of on-chip BSI do not lead to meaningful differences in the fringe pattern seen, as common capillaries have been shown to match theory excellently [14,19]. However, there are many potential advantages to performing BSI using a microfluidic system; for example, more straightforward analysis of the fringe pattern and dechirping, inherent liquid handling, the ability to multiplex the system (including compensation [10]), and the ability to create novel geometries. These factors could prove advantageous when considering the use of BSI as a portable diagnostic device, possibly in conjunction with inexpensive laser sources [24]. Despite this, due to its longer optical path length for a given radius, ease of manufacture, higher fringe modulation depth, and better understood theory, the capillary-based approach still appears to have significant benefits compared to on-chip BSI.

## 6. Conclusions

A comprehensive model of backscattering interferometry utilising a semicircular channel was outlined here, facilitating modelling of the expected interference fringes for both *s*- and *p*-polarised input light at any incident angle. The simulation, based on a previously validated ray tracing approach, was validated and compared to physical principles thoroughly before analysis on the final fringe patterns obtained was performed. It was found that at all incident angles, on-chip BSI leads to a simple, 2-beam fringe pattern with a strong increase in spatial frequency with detection angle. However this spatial frequency increase can be mitigated, resulting in a single frequency component, greatly decreasing analysis complexity. Comparisons of the model presented here with published experimental data were made, and it was found that they did not agree in some areas, highlighting the problems with interpretation of BSI data and the need for greater research into this technique. These discrepancies are most likely due to the difference in geometry between the simulation (purely semicircular) and experiments (approximately semicircular). More specifically, it is hypothesised here that a slope in the extended flat central region in the literature analysed here gives rise to the experimentally observed fringe patterns. Finally, after a comprehensive analysis of on-chip backscattering interferometry, we conclude that for reasons including ease of use, cost-effectiveness, and data quality, capillary-based backscattering interferometry still has significant benefits over on-chip BSI.

## Figures and Tables

**Table 1:** A table showing the number of intersections *i* and the bounds on the values of x/r at which they occur for the case where ψ=0, correct to 3 significant figures. For example, between x/r=0 and x/r=0.5, there will always only be a single intersection between the ray and the channel circumference (e.g., Figure 1A).

Intersection Number, *i*	Bounds on Incident Ray
1	0≤x/r<0.500
2	0.500≤x/r<0.809
3	0.809≤x/r<0.901
4	0.901≤x/r<0.940
5	0.940≤x/r<0.959
6	0.959≤x/r<0.971

## Data Availability

The data presented in this study are available in the Article and Supplementary Information.

## Supplementary Materials

The following are available at https://www.mdpi.com/article/10.3390/s22114301/s1, Equation (S1): General equation for the *x*-coordinate of the first intersection point of a *c*-type ray with the channel perimeter. Figure S1: Simulated fringe patterns for an obliquely illuminated channel (3 degrees).
