# Supplementary material for: An Analysis of Semicircular Channel Backscattering Interferometry through Ray Tracing Simulations
Source: Sensors (Basel). 2022 Jun 6;22(11):4301. doi: 10.3390/s22114301 (PMC9185450; doi:10.3390/s22114301)
Supplement: Supplementary file 1 [file sensors-22-04301-s001.zip › sensors-1758285-supplementary.pdf]

Supplementary Information - An Analysis of Semicircular Channel Backscattering Interferometry Through Ray Tracing Simulations

Niall M. C. Mulkerns,[†,‡] William H. Hoffmann,[†,‡,¶] Ian D. Lindsay,[†,‡] and Henkjan Gersen[*,†,‡]

†*H. H. Wills Physics Laboratory, University of Bristol, UK*

‡*Bristol Centre for Functional Nanomaterials, University of Bristol, UK*

¶*School of Chemistry, University of Bristol, UK*

E-mail: h.gersen@bristol.ac.uk

Unless otherwise explicitly stated, all variables within the Supporting Information are as defined within the main body of the text.

General Channel Intersection Position Formula

The x position of the first intersection of the ray with the radius of the channel x_θ in the general case of $\psi \neq 0$ can be written

$$x_\theta = \cos^2(\epsilon)\left(-t\tan(\omega) - x \pm \tan(\epsilon)\sqrt{r^2\tan^2(\epsilon) + r^2 - t^2\tan^2(\omega) - 2tx\tan(\omega) - x^2}\right), \tag{S1}$$

by rearranging Eq. 2, which was solved using the symbolic mathematics package `sympy` (version 1.1.1) in Python3. In this work, the negative root was used

Oblique Fringe Pattern

Fig. S1 shows the observed fringe pattern at the detector plane for an incident angle of $\psi = 3°$, with all other parameters as defined in the text. The fringes bear a high degree of similarity to those in Fig. 5, with a small reduction in the overall intensity of the p-polarised case. This is due to the fact that as the angle increases, the magnitude of the reflection coefficient decreases for p-polarised light, but increases for s-polarised light, causing small discrepancies in the fringe intensities.

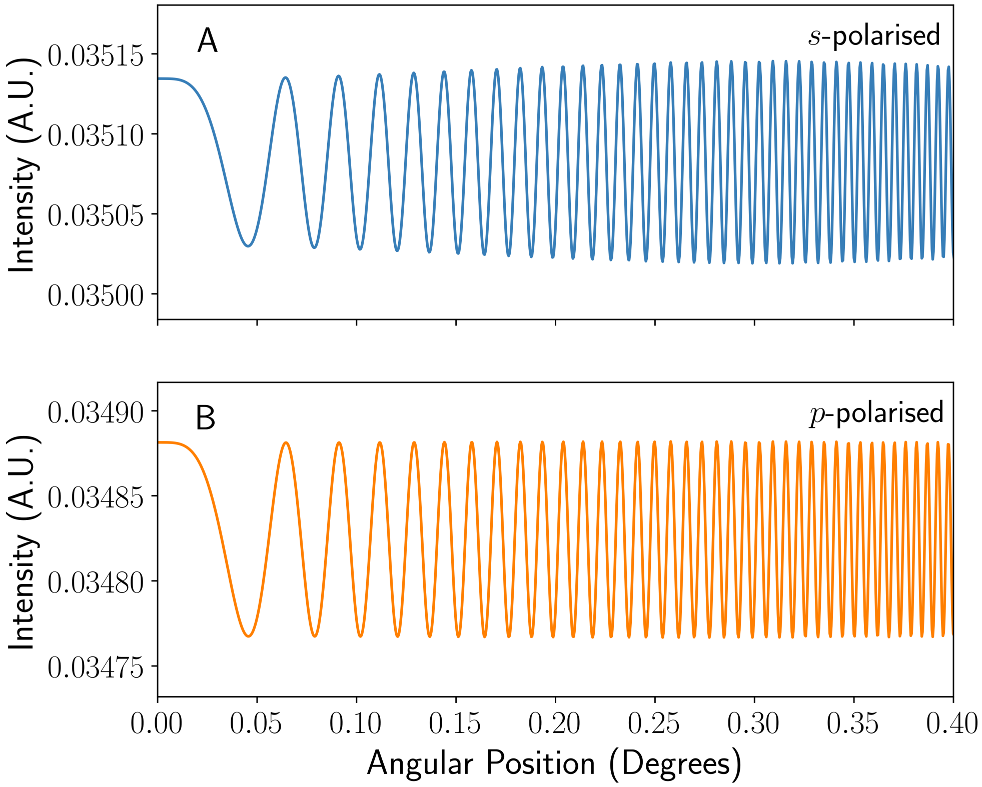

Figure S1: Graphs showing the interference patterns seen on a detector for an incident angle of $\psi = 3°$ using the parameters as set out in the main body of text at a distance of 1 m with the angle given from the line of $x = 0$. A shows the interference pattern for s-polarised incident light, whereas B shows the pattern imaged for p-polarised light.
